# Sequential Multiple Imputation for Real-World Health-Related Quality of Life Missing Data after Bariatric Surgery

**DOI:** 10.3390/ijerph191710827

**Published:** 2022-08-30

**Authors:** Sun Sun, Nan Luo, Erik Stenberg, Lars Lindholm, Klas-Göran Sahlén, Karl A. Franklin, Yang Cao

**Affiliations:** 1 Department of Epidemiology and Global Health, Umeå University, 901 87 Umeå, Sweden; 2 Research Group Health Outcomes and Economic Evaluation, Department of Learning, Informatics, Management and Ethics, Karolinska Institutet, 171 77 Stockholm, Sweden; 3 Saw Swee Hock School of Public Health, National University of Singapore, Singapore 117549, Singapore; 4 Department of Surgery, Faculty of Medicine and Health, Örebro University, 701 85 Örebro, Sweden; 5 Department of Surgical and Perioperative Sciences, Surgery, Umeå University, 901 87 Umeå, Sweden; 6 Clinical Epidemiology and Biostatistics, School of Medical Sciences, Örebro University, 701 82 Örebro, Sweden

**Keywords:** multiple imputations, health-related quality of life, SF-36, health utility, real-world data

## Abstract

One of the main challenges for the successful implementation of health-related quality of life (HRQoL) assessments is missing data. The current study examined the feasibility and validity of a sequential multiple imputation (MI) method to deal with missing values in the longitudinal HRQoL data from the Scandinavian Obesity Surgery Registry. All patients in the SOReg who received bariatric surgery between 1 January 2011 and 31 March 2019 (*n* = 47,653) were included for the descriptive analysis and missingness pattern exploration. The patients who had completed the short-form 36 (SF-36) at baseline (year 0), and one-, two-, and five-year follow-ups were included (*n* = 3957) for the missingness pattern simulation and the sequential MI analysis. Eleven items of the SF-36 were selected to create the six domains of SF-6D, and the SF-6D utility index of each patient was calculated accordingly. The multiply-imputed variables in previous year were used as input to impute the missing values in later years. The performance of the sequential MI was evaluated by comparing the actual values with the imputed values of the selected SF-36 items and index at all four time points. At the baseline and year 1, where missing proportions were about 20% and 40%, respectively, there were no statistically significant discrepancies between the distributions of the actual and imputed responses (all *p*-values > 0.05). In year 2, where the missing proportion was about 60%, distributions of the actual and imputed responses were consistent in 9 of the 11 SF-36 items. However, in year 5, where the missing proportion was about 80%, no consistency was found between the actual and imputed responses in any of the SF-36 items. Relatively high missing proportions in HRQoL data are common in clinical registries, which brings a challenge to analyzing the HRQoL of longitudinal cohorts. The experimental sequential multiple imputation method adopted in the current study might be an ideal strategy for handling missing data (even though the follow-up survey had a missing proportion of 60%), avoiding significant information waste in the multivariate analysis. However, the imputations for data with higher missing proportions warrant more research.

## 1. Introduction

Health-related quality of life (HRQoL) represents the subjective evaluation of a patient’s health status, providing complementary information to survival, cures, and biological responses to treatment [1]. HRQoL data have been increasingly collected in clinical trials, population health surveys, and clinical registers in many countries [2,3,4,5]. However, one of the main challenges for the successful implementation of HRQoL assessments is missing data, which can be at the item level, i.e., respondents do not provide answers to certain items in an HRQoL questionnaire, or missing entire forms due to loss of follow-ups [1]. The missing data may lead to biased conclusions if unattended. Therefore, it is important to understand missingness patterns and handle missing data properly when analyzing HRQoL data. In economic evaluations, HRQoL instruments that can be used to generate health utility data, also known as preference-based measures, are applied. The most commonly applied preference-based measures are EQ-5D [6], short-form-6D (SF-6D) [7,8], and the health utilities index [9]. Missing data are also important issues when it comes to health utility calculations since these measures require complete answers to all of the relevant items [1,10].

Both missing items and missing forms in HRQoL are rather common in clinical trials or observational studies, which may reduce statistical power and present a challenge for research in this field [11,12]. During the data collection phases, strategies could be integrated into the study design to minimize the incidence of missing data. However, once the trial/registry has started, as an analyst, one has little influence on how data are collected but primarily relies on analytical methods to account for missing data [13]. The development of sound strategies for handling missing data includes imputation methods. The current practices of handling missing data in HRQoL studies include list-wise deletion, single imputation by replacing the missing value with one previously observed value or mean value, multiple imputation(s) (MI), and model-based approaches [14,15]. Among them, MI methods are widely recommended because they may incorporate uncertainty around the missing values [16]. However, this is often poorly applied in reality [17].

Managing missing real-world data, including HRQoL data, has become a challenging issue with the rapidly increasing applications to real-world data in recent years. Real-world data are derived from a number of sources that document outcomes in a heterogeneous patient population in real-world settings, including (but not limited to) electronic health records, health insurance claims, and patient surveys [18]. Real-world data provide insights beyond those that can be derived from clinical trials as they follow patients with different characteristics in real-life situations, and often for longer periods than clinical trials [19]. Compared with well-conducted randomized controlled trials (RCTs), missing data are more pronounced in real-world data because data can be missing for exposures, known confounders, and outcomes [13]. However, existing guidance and standards for handling missing data most often only concern RCTs. Currently, there are no standards or formal guidelines on how to deal with missing real-world HRQoL data [13].

In this study, we demonstrated and simulated the missingness of real-world HRQoL data from the Scandinavian Obesity Surgery Registry (SOReg) [20], and examined whether a sequential MI procedure is a practical strategy for handling missing values in the short-form 36 (SF-36) and SF-6D forms. Because the HRQoL data were repeatedly collected at four time points, the sequential MI procedure imputed the missing values chronologically, i.e., the missing data in a later follow-up were imputed using the multiply-imputed datasets of a prior follow-up.

## 2. Materials and Methods

This research applied data from existing registers in Sweden. Data retrieval, analyses, and presentation results were performed in accordance with the Declaration of Helsinki. The research work was approved by the Swedish Ethical Review Agency (Etikprövningsmyndigheten; approval numbers: 2019-03666 and 2019-05713).

### 2.1. Data Sources

The Scandinavian Obesity Surgery Registry (SOReg) is a Swedish national quality registry for bariatric surgery management and research. It has a coverage of >98% nationwide, its internal validity is evaluated regularly, and it has high data quality [21]. Data on patients’ sociodemographic information, hospital characteristics, and detailed information regarding the surgeries and post-surgery outcomes, including HRQoL assessed by SF-36 and the Obesity Problem Scale [22,23], were obtained from the SOReg. Patients included in the study reported their HRQoL data at baseline (i.e., prior surgery) and years 1, 2, and 5 postoperatively by filling out a questionnaire. Specialized nurses collected anthropometric data and completed questionnaires. Data entry was performed by trained persons (participating surgeons, plus dedicated nurses in each center).

In the current study, all patients who received bariatric surgery between 1 January 2011 and 31 March 2019 (*n* = 47,653) were included for descriptive analysis and missingness pattern explorations, while only patients who had completed SF-36 at baseline (year zero), and at the one-, two-, and five-year follow-ups were included (*n* = 3957) as part of an analytical dataset to evaluate the multiple imputation process.

### 2.2. SF-36 and SF-6D

SF-36 measures HRQoL with 36 items, which can be grouped into 8 domains (physical function, role—physical, bodily pain, general health, vitality, social function, role-emotional, and mental health), and each item contains 2–6 severity levels [24]. In order to elicit the health utility, 11 items of the SF-36 (Supplementary Material Table S1) were selected to create SF-6D, including 6 domains (pain, mental health, physical functioning, social functioning, role participation, and vitality). Each domain described four to six severity levels (Supplementary Material Table S2) [7]. The SF-6D utility index of each patient in the current study was calculated using the Formula (1) below:

(1) $$SF-6D_{index}=1-(\beta_{constant}+\overset{6}{\underset{i=1}{\sum }}\overset{5\;or\;6}{\underset{m=2}{\sum }}\beta_{yi=mX_{yi}=m\;}+\beta_{Most}Most)$$

where *yi* (*i* = 1, 2, …, 6) indicates SF-6D domains that can take *m* levels (*m* = 2, 3, …, 5 or 6); *X_yi_* = *m* represents dummy variables that indicate levels 2 to 5 or 6 and $\beta_{yi=mX_{yi}=m}$ is the associated coefficient; $\beta_{constant}$ corresponds to the constant deviating from full health; and *Most* is a dummy variable, indicating that there is at least one dimension at levels 5 or 6.

Missing information on any of the 11 items would lead to missingness in the SF-6D domains (the right hand of the equation), which in turn would lead to missingness in the SF-6D index score. There were two methods applied for index imputation: (1) to impute the items, firstly, then calculate the index based on the above formula; (2) to impute the index directly. There might be differences in the results when the two different methods are used. In the current study, we applied the second method, as it is also useful even when information from the items is missing.

### 2.3. Missingness Mechanism and Missingness Pattern Simulation

The widely used missingness mechanisms in simulation studies on multiple imputations are: missing completely at random, missing at random (MAR), and missing not at random [16]. In the current study, we simulated missingness in the analytical dataset according to a MAR mechanism, which assumes that the probability of the data that are missing does not depend on the unobserved data, but is conditional on the observed data.

**Figure 1:**
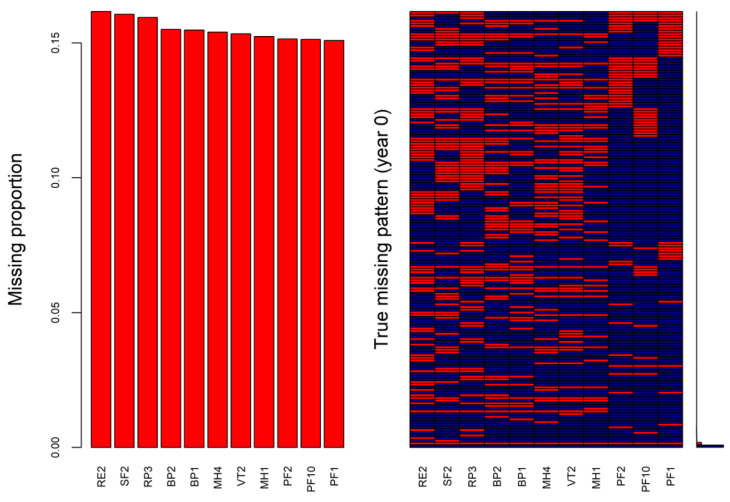
Missing proportions (**left**) and missingness patterns (**right**) of the selected 11 SF-36 items at baseline in the real-world data (red cells indicating missing). BP, bodily pain; MH, mental health; PF, physical function; RE, role-emotional; RP, role participation; SF, social function; VT, vitality.

To ensure that the missingness patterns of the analytical dataset used for multiple imputations may reflect the patterns found in the real-world data, we explored missingness patterns of the selected 11 SF-36 items and SF-6D index at baseline and one-, two-, and five-year follow-ups for the real-world data. The missingness pattern explorations were conducted using the package *mice* in the statistical software R 4.1.1 (R Foundation for Statistical Computing, Vienna, Austria). The overall missing proportion of the SF-36 items at baseline was 19.6%. The missing proportions of the 11 SF-36 items at baseline from the highest to the lowest are shown in Figure 1 (left) and a total of 163 missingness patterns were found (Figure 1, right). Each row in the right panel of Figure 1 is a missingness pattern that indicates where the missing values (red colored) are located in the 11 SF-36 items.

The overall missing proportions of the 11 SF-36 items in the one-, two-, and five-year follow-ups were 40.7%, 62.9%, and 83.8%, respectively, and the missingness patterns are shown in Supplemental Figures S1–S3. In total, 150, 105, and 64 missingness patterns of the 11 SF-36 items were found in the one-, two-, and five-year follow-ups, respectively.

To evaluate the performance of the proposed MI procedure using the analytical dataset, there was a need to simulate the missingness in the data by masking some known values in the analytical dataset. The missingness patterns (right panels of Figure 1, Supplemental Figures S1–S3) detected in the real-world data were applied to mask the values in the 11 SF-36 items at the four time points of the analytical dataset. The masking of the known values was conducted using the package *mice* as well.

**Figure 2:**
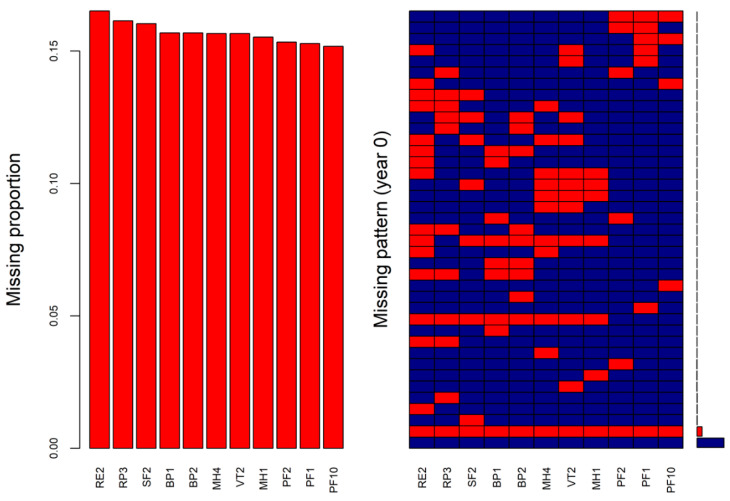
Simulated missing proportions (**left**) and missingness patterns (**right**) of the selected 11 SF-36 items at baseline in the analytical dataset (red cells indicating missing).

The simulated missingness of the analytical dataset for the selected 11 SF-36 items at baseline is shown in Figure 2, with an overall missing proportion of 20.0%. The missing proportions of the 11 items in the analytical dataset (Figure 2, left) were similar to those in the real-world data (Figure 1, left). The simulated missingness of the analytical dataset for the selected 11 SF-36 items in the one-, two-, and five-year follow-ups are shown in Supplemental Figures S4–S6, with the overall missing proportions of 40.4%, 63.8%, and 83.7%, respectively.

In general, the number of missing patterns decreased with reduced observations. Because the analytical dataset has much fewer observations than those in the real-world data (3957 vs. 47,653), the missingness patterns of the analytical dataset were less than those of the real-world data; however, the percentages of the top missingness patterns of both datasets were similar.

**Figure 3:**
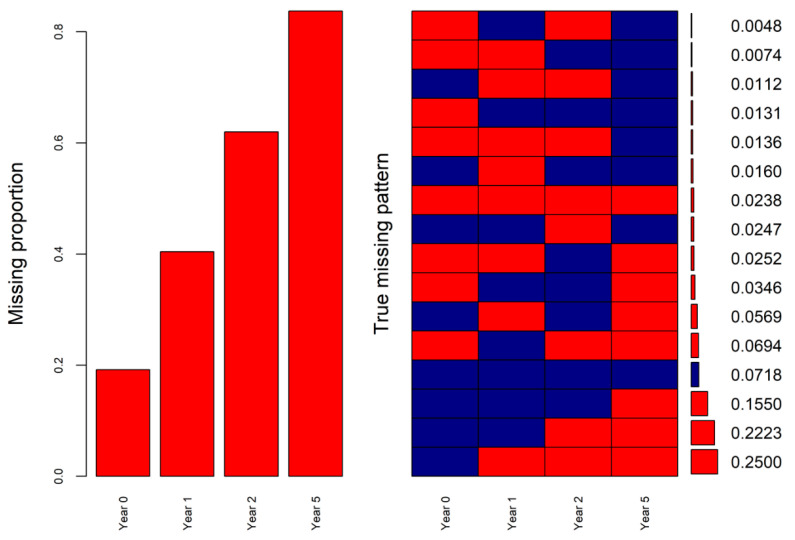
Missing proportions (**left**) and missingness patterns (**right**) of the SF-6D index at four time points in the real-world data (red cells indicating missing).

**Figure 4:**
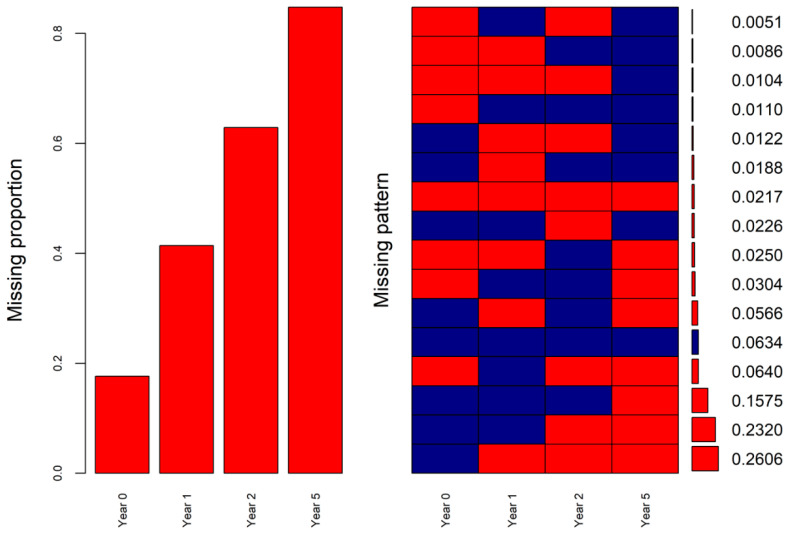
Simulated missingness patterns of SF-6D index at four time points in the analytical dataset (red cells indicating missing).

The missingness patterns of the SF-6D index at the four time points were also obtained from real-world data. The masking of the known SF-6D index values in the analytical dataset was conducted according to the patterns. The missing proportions and missingness patterns of the SF-6D index at baseline (year 0) and one-, two-, and five-year follow-ups in the real-world data and the analytical dataset are shown in Figure 3 and Figure 4. The missing proportions and percentages of the top missingness patterns of both datasets were similar (Figure 3 and Figure 4).

### 2.4. Process of the Sequential Multiple Imputation

**Figure 5:**
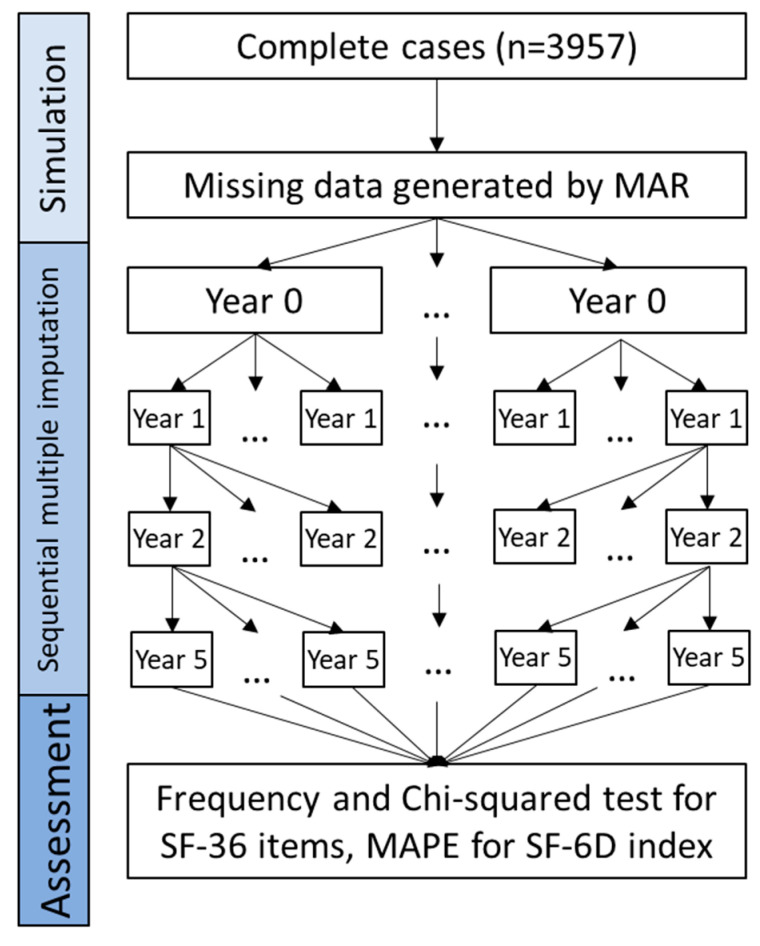
Flowchart of simulation, sequential multiple imputation, and assessment. MAPE, mean absolute percentage error.

We applied a sequential method to impute the missing values at baseline (year 0), and years 1, 2, and 5 in order. The process of the sequential multiple imputation is shown in Figure 5, the “Sequential multiple imputation” step, and described in detail as follows:

Firstly, the missing values of the selected 11 SF-36 items at baseline (year 0) were multiply-imputed (five imputations were used in the current study) using all baseline variables, including age, sex, BMI, pregnancy, and comorbidities, including sleep apnea, hypertension, diabetes, dyslipidemia, dyspepsia, diarrhea, depression, and other illnesses that may have contributed to the surgical decisions. Five imputed datasets were generated for the baseline data.

Secondly, for each imputed baseline dataset, the missing values of the selected 11 SF-36 items and comorbidities at the one-year follow-up were multiply-imputed based on all the baseline variables, as well as the previously imputed SF-36 items. Five imputed datasets were generated for each imputed baseline (year 0) dataset; therefore, in total, 5 × 5 = 25 imputed datasets were generated for the one-year follow-up.

Similarly, for missing values in the two- and five-year follow-ups, they were imputed five times for each previously imputed dataset based on all the variables in the previous years. Therefore, in total, 125 (5 × 25 imputed datasets of year 1) and 625 (5 × 125 imputed datasets of year 2) imputed datasets were generated for the two- and five-year follow-ups, respectively.

When conducting the multiple imputations within each year, the multivariate imputation using chained equations was used, with predictive mean matching, logistic regression, and proportional odds regression for continuous, binary, and ordered variables, respectively [25].

### 2.5. Assessment of Performance

The performance of the sequential multiple imputation approach was evaluated by comparing the actual values with the imputed values of the selected 11 SF-36 items and index at all four time points (baseline, one-, two-, and five-year follow-ups). The SF-36 items were compared using frequency distributions of the actual and imputed item scores, and the agreement of the distributions was tested using the chi-squared test controlled for the false discovery rate [26,27]. The mean absolute percentage error (MAPE), one of the most common metrics used to measure accuracy for continuous variables, was calculated to assess the agreement between the actual and imputed values for the SF-6D index [28,29,30]. MAPE is the mean of the absolute difference between the actual and imputed values divided by the actual values [31]. MAPE < 10% is excellent, <20% is good, 20–50% is fine, and >50% is poor [32].

The intraclass correlation coefficients (ICCs) were also provided to indicate the agreement between the actual values and the imputed values. An ICC value below 0.50, between 0.50 and 0.75, between 0.75 and 0.90, or above 0.90 indicates poor, moderate, good, or excellent agreement, respectively [33].

In the current study, MAPE and ICC were averaged across the imputations.

All statistical analyses were conducted in R 4.11 (R Foundation for Statistical Computing, Vienna, Austria) and Stata 17.0 (College Station, Texas, USA). A two-sided *p*-value < 0.05 was considered statistically significant.

## 3. Results

### 3.1. Characteristics of the Patients

The demographic characteristics of the patients at baseline are shown in Table 1. Statistically significant differences were found in most variables between the patients included in the analytical dataset and those excluded (with at least one missing form). In general, the patients in the analytical dataset were older and fewer (in proportion) of them had comorbidities.

Descriptive analysis of the selected 11 SF-36 items and SF-6D index at baseline are shown in Table 2. Similarly, statistically significant differences in proportions of the SF-36 item scores and mean values of the SF-6D index were found between the patients included and excluded. In general, the respondents in the analytical dataset reported much fewer missing items and a slightly higher SF-6D index, compared to those excluded.

Demographics and comorbidities, SF-36 items scores, and SF-6D indices in one-, two-, and five-year follow-ups are shown in Supplemental Tables S3–S8. At all three time points, the included respondents had much fewer missing values on characteristics and SF-36 items, and were relatively healthier, compared to those excluded.

### 3.2. Imputation Results for the Selected SF-36 Items

The comparisons of distributions between the actual and imputed responses of the patients in the analytical dataset are shown in Table 3. At the baseline and year 1, where missing proportions were about 20% and 40%, respectively, there were no statistically significant discrepancies between the distributions of the actual and imputed responses (all *p*-values > 0.05). In year 2, where the missing proportion was about 60%, distributions of the actual and imputed responses were consistent in most SF-36 items, except for PF2 and PF10. However, in year 5, where the missing proportion rose to about 80%, no consistency was found between the actual and imputed responses in any of the SF-36 items. The results indicate that the imputation based on the previous demographic and comorbidity information works well for the SF-36 items even when the missing proportion was as high as 60%. According to the ICC values presented in Table 3, we can see that the agreements between the actual values and the imputed values of the SF-36 items were good at baseline and in year 1 but moderate and poor in years 2 and 5, respectively.

### 3.3. Imputation Results for SF-6D Index

In general, the imputed SF-6D index values had similar means and standard errors as the actual ones (Table 4). For the baseline, the MAPE of the imputed index was smaller than 5% of the actual index, which means, on average, the imputed index values ranged between 95% and 105% of the actual value. Even for the follow-up in postoperative year 2, the deviation of the imputed index values from the actual values was smaller than 10% (Table 4). The results indicate that the imputation method may provide relatively accurate values for the continuous index in terms of the mean absolute percentage error when the missing proportion is around 60%. According to the ICC values presented in Table 4, we can see that the agreement between the actual and imputed values of the SF-6D index was good at baseline but moderate in the one-, two-, and five-year follow-ups.

## 4. Discussion

Real-world data collections, compared to RCTs, face more challenges. Firstly, a high proportion of missing forms is possible due to long follow-ups, especially when the follow-up time extends beyond two years [11,12]. Moreover, the follow-up time points of most clinical registers are based on the need for care and are not standardized, which brings additional challenges in handling data missingness and analysis [34]. Secondly, missing or incompleteness in confounder measures in real-world data are more common compared to clinical trials, which might distort the inference. For example, patients lost to follow-ups might be due to characteristics that cannot be randomized, such as deteriorating health, which would introduce bias in MI and later inferential statistics. However, almost all of the guidelines regarding how to handle missingness with HRQoL data are for clinical trials only [13,14,35]. Therefore, our study might contribute to the development of guidance for good practices for the prevention and handling of missing data in real-world HRQoL data.

### 4.1. Main Findings

In the current study, we explored the missing data problem in the HRQoL data of a clinical register (SOReg) and examined a sequential multiple imputation method as a potential solution for the missing item and form problem in the repeated data collection using the SF-36 questionnaire. To the best of our knowledge, this was the first time that the sequential multiple imputation method was applied to impute HRQoL data. This method is preferred as it takes into consideration the longitudinal nature of HRQoL data collected in clinical studies; that is, a patient’s HRQoL at follow-ups is determined by his/her previous HRQoL i.e., at the baseline and previous follow-ups. Although the missing proportion was high for the self-reported HRQoL questionnaire, the sequential multiple imputation method may still provide quite similar distributions for the dataset with missing values even when the missing proportion is as high as 60%. The method has provided a potential solution to handle missing data for multivariable analyses in HRQoL studies when missing was quite substantial.

Estimation of the SF-6D index requires complete answers on all 11 items from SF-36 or SF-12 [7,36]. Knowledge of missingness patterns, especially items associated with high missing proportions, is crucial to prevent missing data and select appropriate imputation methods. Knowledge of missingness patterns ((in terms of which characteristics of patients and providers are associated with missing data) might enable one to use appropriate strategies to reduce missing data during the process of data collection [12]. In the current study, although we found that there were many different combinations in missingness patterns for SF-6D, there was no dominating pattern, suggesting that the missingness on the 11 items used for SF-6D were independent of each other. 

The overall missing proportions of the 11 SF-6D items increased over time, which might suggest that the ‘missing’ is associated with the extension of the follow-up. Based on the missing at random mechanism of the 11 items, the sequential multiple imputation method achieved satisfactory agreement between the actual data and the imputed data even when the missing proportion was as high as 63% at the two-year follow-up. However, as expected, the imputation could not approximate the actual data at the five-year follow-up where the missing proportion was >80%. One reason for the worse performance of the sequential MI procedure in the two- and five-year follow-ups might be the effect of propagation of uncertainty (or propagation of error) embedded in the procedure [37]. Because the MI for a particular year has already incorporated uncertainty regarding the missing data of the year, the sequential MI for data in later years could propagate the uncertainty due to the uncertainty of the parameters in the function for imputation, which are inherited from the previously multiply-imputed data [38].

### 4.2. Strengths and Limitations

In the current study, we adopted and evaluated the sequential multiple imputation method for four waves of HRQoL data collected from around four thousand patients in a registry. Although the performance of the items and index cannot be compared directly in our study, both imputations presented a high agreement between the imputed data and the real-world data when the missing proportion was < 50%, showing great application potential. We hope our study may stimulate more research on missingness in real-world HRQoL data. Efficient imputation methods would help improve the translation of HRQoL data into complete, accurate, and reliable evidence for healthcare decision-making [13].

There are limitations in the current study. Firstly, because the actual values of the missing SF-36 items and SF-6D index in the real-world data were unknown, it was impossible to identify the mechanism of the missingness in the current study; therefore, we applied the missing at random mechanism for the missing values. However, if the probability of missingness for an item was dependent on what would have been true or the item’s non-response was ‘missing not at random’, for example, patients with worse health were more likely to have missing HRQoL items and/or forms, the current multiple imputation method would not be sufficient and other missing data models with different assumptions should be investigated [39]. Secondly, the proposed sequential MI and its performance were evaluated based on the simulation using the complete respondents, i.e., those who had all four HRQoL forms during the 5-year follow-up. However, we observed statistically significant differences between the patients with missing forms and complete forms in the current study. Although the differences were minor and the statistical significance might have been due to the large sample size, a possible bias introduced by the imputation based on the data of the complete respondents cannot be ruled out. Thirdly, in the current study, we investigated missing data concerning SF-36 and SF-6D in a clinical registry for obese patients. Further investigations on missingness based on other HRQoL instruments, such as EQ-5D, the Health Utility Index, and other patient groups, are also needed. It might be that HRQoL instruments with more items are more likely associated with higher missing proportions, which should be considered when designing the data collection strategy.

## 5. Conclusions

Relatively high missing proportions in HRQoL data—especially after long-term follow-ups—are common in clinical registries, which brings challenges to analyzing the HRQoL of longitudinal cohorts. The sequential multiple imputation method adopted in the current study might provide an ideal imputation for the missing data (even though the follow-up survey had a missing proportion of 60%), avoiding significant information waste in the multivariable analysis. However, imputations for data with higher missing proportions (above 60%) are unclear. To prevent and handle the missing data in HRQoL studies, researchers should apply a rigorous methodology and practices. Guidance for preventing and handling missing data in observational studies is needed, and studies that use real-world data should be prioritized.

## Figures and Tables

**Table 1:** Demographic characteristics of the patients at baseline.

Variable		All	Excluded	Analytical Dataset	*p*-Value *
N		46,753	42,796	3957	
Age (mean (SD))		41.06 (11.30)	40.75 (11.28)	43.82 (11.15)	<0.001
BMI (mean (SD))		41.57 (5.65)	41.53 (5.66)	42.01 (5.48)	<0.001
Sex (%)	Man	10,933 (23.4)	10,058 (23.5)	875 (22.1)	0.050
	Woman	35,820 (76.6)	32,738 (76.5)	3082 (77.9)	
Smoking (%)	No	28,781 (61.6)	26,402 (61.7)	2379 (60.1)	0.067
	Yes	4765 (10.2)	4381 (10.2)	384 (9.7)	
	Quit	8075 (17.3)	7355 (17.2)	720 (18.2)	
	Missing	5132 (11.0)	4658 (10.9)	474 (12.0)	
Pregnancy (%)	No	39,862 (85.3)	35,905 (83.9)	3957 (100.0)	<0.001
	Missing	6891 (14.7)	6891 (16.1)	0 (0.0)	
Comorbidity (%)	No	17,620 (37.7)	15,869 (37.1)	1751 (44.3)	<0.001
	Yes	22,240 (47.6)	20,034 (46.8)	2206 (55.7)	
	Missing	6893 (14.7)	6893 (16.1)	0 (0.0)	
Sleep apnea (%)	No	35,749 (76.5)	32,245 (75.3)	3504 (88.6)	<0.001
	Yes	4111 (8.8)	3658 (8.5)	453 (11.4)	
	Missing	6893 (14.7)	6893 (16.1)	0 (0.0)	
Hypertension (%)	No	29,890 (63.9)	27,161 (63.5)	2729 (69.0)	<0.001
	Yes	9970 (21.3)	8742 (20.4)	1228 (31.0)	
	Missing	6893 (14.7)	6893 (16.1)	0 (0.0)	
Diabetes (%)	No	34,668 (74.2)	31,305 (73.1)	3363 (85.0)	<0.001
	Yes	5192 (11.1)	4598 (10.7)	594 (15.0)	
	Missing	6893 (14.7)	6893 (16.1)	0 (0.0)	
Dyslipidemia (%)	No	36,018 (77.0)	32,555 (76.1)	3463 (87.5)	<0.001
	Yes	3842 (8.2)	3348 (7.8)	494 (12.5)	
	Missing	6893 (14.7)	6893 (16.1)	0 (0.0)	
Dyspepsia (%)	No	35,564 (76.1)	32,037 (74.9)	3527 (89.1)	<0.001
	Yes	4296 (9.2)	3866 (9.0)	430 (10.9)	
	Missing	6893 (14.7)	6893 (16.1)	0 (0.0)	
Diarrhea (%)	No	39,213 (83.9)	35,339 (82.6)	3874 (97.9)	<0.001
	Yes	647 (1.4)	564 (1.3)	83 (2.1)	
	Missing	6893 (14.7)	6893 (16.1)	0 (0.0)	
Depression (%)	No	33,355 (71.3)	29,904 (69.9)	3451 (87.2)	<0.001
	Yes	6505 (13.9)	5999 (14.0)	506 (12.8)	
	Missing	6893 (14.7)	6893 (16.1)	0 (0.0)	
Other illness (%)	No	35,519 (76.0)	31,967 (74.7)	3552 (89.8)	<0.001
	Yes	4343 (9.3)	3938 (9.2)	405 (10.2)	
	Missing	6891 (14.7)	6891 (16.1)	0 (0.0)	
Obesity problem summary score (mean (SD))		65.06 (26.11)	65.62 (25.98)	60.05 (26.73)	<0.001

* Student’s *t*-test was used to compare the means and the chi-squared test was used to compare percentages.

**Table 2:** Scores for the selected SF-36 items and SF-6D index at baseline.

SF-6D Item	Level	All (N = 46,753)	Excluded (N = 42,796)	Analytical Dataset (N = 3957)	*p*-Value *
PF1 (%)	1	26,464 (56.6)	23,794 (55.6)	2670 (67.5)	<0.001
	2	11,523 (24.6)	10,388 (24.3)	1135 (28.7)	
	3	1712 (3.7)	1576 (3.7)	136 (3.4)	
	Missing	7054 (15.1)	7038 (16.4)	16 (0.4)	
PF2 (%)	1	4878 (10.4)	4445 (10.4)	433 (10.9)	<0.001
	2	22,027 (47.1)	19,875 (46.4)	2152 (54.4)	
	3	12,767 (27.3)	11,417 (26.7)	1350 (34.1)	
	Missing	7081 (15.1)	7059 (16.5)	22 (0.6)	
PF10 (%)	1	2853 (6.1)	2656 (6.2)	197 (5.0)	<0.001
	2	13,910 (29.8)	12,576 (29.4)	1334 (33.7)	
	3	22,917 (49.0)	20,507 (47.9)	2410 (60.9)	
	Missing	7073 (15.1)	7057 (16.5)	16 (0.4)	
RP3 (%)	1	17,956 (38.4)	16,392 (38.3)	1564 (39.5)	<0.001
	2	21,344 (45.7)	18,999 (44.4)	2345 (59.3)	
	Missing	7453 (15.9)	7405 (17.3)	48 (1.2)	
RE2 (%)	1	14,899 (31.9)	13,706 (32.0)	1193 (30.1)	<0.001
	2	24,297 (52.0)	21,595 (50.5)	2702 (68.3)	
	Missing	7557 (16.2)	7495 (17.5)	62 (1.6)	
SF2 (%)	1	1503 (3.2)	1421 (3.3)	82 (2.1)	<0.001
	2	4393 (9.4)	4077 (9.5)	316 (8.0)	
	3	8624 (18.4)	7890 (18.4)	734 (18.5)	
	4	9278 (19.8)	8382 (19.6)	896 (22.6)	
	5	15,447 (33.0)	13,574 (31.7)	1873 (47.3)	
	Missing	7508 (16.1)	7452 (17.4)	56 (1.4)	
BP1 (%)	1	5669 (12.1)	5068 (11.8)	601 (15.2)	<0.001
	2	4777 (10.2)	4232 (9.9)	545 (13.8)	
	3	6042 (12.9)	5416 (12.7)	626 (15.8)	
	4	14,001 (29.9)	12,606 (29.5)	1395 (35.3)	
	5	7103 (15.2)	6472 (15.1)	631 (15.9)	
	6	1927 (4.1)	1801 (4.2)	126 (3.2)	
	Missing	7234 (15.5)	7201 (16.8)	33 (0.8)	
BP2 (%)	1	10,196 (21.8)	9045 (21.1)	1151 (29.1)	<0.001
	2	9781 (20.9)	8767 (20.5)	1014 (25.6)	
	3	10,232 (21.9)	9233 (21.6)	999 (25.2)	
	4	6772 (14.5)	6202 (14.5)	570 (14.4)	
	5	2526 (5.4)	2343 (5.5)	183 (4.6)	
	Missing	7246 (15.5)	7206 (16.8)	40 (1.0)	
MH1 (%)	1	779 (1.7)	731 (1.7)	48 (1.2)	<0.001
	2	1788 (3.8)	1684 (3.9)	104 (2.6)	
	3	3688 (7.9)	3411 (8.0)	277 (7.0)	
	4	6184 (13.2)	5644 (13.2)	540 (13.6)	
	5	11,644 (24.9)	10,466 (24.5)	1178 (29.8)	
	6	15,548 (33.3)	13,764 (32.2)	1784 (45.1)	
	Missing	7122 (15.2)	7096 (16.6)	26 (0.7)	
MH4 (%)	1	797 (1.7)	747 (1.7)	50 (1.3)	<0.001
	2	2025 (4.3)	1903 (4.4)	122 (3.1)	
	3	3554 (7.6)	3284 (7.7)	270 (6.8)	
	4	6072 (13.0)	5553 (13.0)	519 (13.1)	
	5	13,164 (28.2)	11,822 (27.6)	1342 (33.9)	
	6	13,942 (29.8)	12,323 (28.8)	1619 (40.9)	
	Missing	7199 (15.4)	7164 (16.7)	35 (0.9)	
VT2 (%)	1	844 (1.8)	738 (1.7)	106 (2.7)	<0.001
	2	3573 (7.6)	3138 (7.3)	435 (11.0)	
	3	6083 (13.0)	5408 (12.6)	675 (17.1)	
	4	9448 (20.2)	8409 (19.6)	1039 (26.3)	
	5	11,734 (25.1)	10,697 (25.0)	1037 (26.2)	
	6	7903 (16.9)	7265 (17.0)	638 (16.1)	
	Missing	7168 (15.3)	7141 (16.7)	27 (0.7)	
Index (mean (SD))		0.66 (0.13)	0.66 (0.13)	0.69 (0.13)	<0.001

* Student’s *t*-test was used to compare the means and the chi-squared test was used to compare percentages. BP, bodily pain; MH, mental health; PF, physical function; RE, role-emotional; RP, role participation; SF, social function; VT, vitality.

**Table 3:** Accuracy of multiple imputations for the selected SF-36 item scores.

Items	Score	Year 0		Year 1		Year 2		Year 5	
		Actual	Imputed	Actual	Imputed	Actual	Imputed	Actual	Imputed
PF1	1	2584	2543	460	455	502	543	751	1341
	2	1106	1148	1573	1560	1412	1458	1511	1267
	3	132	144	1778	1809	1892	1833	1559	1226
		χ^2^ = 1.610	*p* = 0.447	χ^2^ = 0.229	*p* = 0.892	χ^2^ = 3.178	*p* = 0.204	χ^2^ = 226.622	*p* < 0.001
		ICC = 0.868	*p* < 0.001	ICC = 0.782	*p* < 0.001	ICC = 0.671	*p* < 0.001	ICC = 0.391	*p* < 0.001
PF2	1	416	390	113	121	109	147	162	940
	2	2086	2088	489	490	534	556	709	546
	3	1315	1356	3229	3223	3175	3131	2952	2348
		χ^2^ = 1.431	*p* = 0.489	χ^2^ = 0.279	*p* = 0.870	χ^2^ = 6.358	*p* = 0.042	χ^2^ = 639.249	*p* < 0.001
		ICC = 0.868	*p* < 0.001	ICC = 0.772	*p* < 0.001	ICC = 0.623	*p* < 0.001	ICC = 0.101	*p* < 0.001
PF10	1	190	170	53	68	73	142	85	754
	2	1295	1278	215	236	293	357	371	421
	3	2334	2386	3558	3530	3460	3335	3368	2659
		χ^2^ = 1.767	*p* = 0.413	χ^2^ = 2.940	*p* = 0.230	χ^2^ = 30.737	*p* < 0.001	χ^2^ = 619.995	*p* < 0.001
		ICC = 0.848	*p* < 0.001	ICC = 0.745	*p* < 0.001	ICC = 0.558	*p* < 0.001	ICC = 0.048	*p* = 0.002
RP3	1	1521	1516	462	465	547	614	775	1600
	2	2279	2318	3344	3369	3255	3220	3022	2334
		χ^2^ = 0.168	*p* = 0.682	χ^2^ = 0.000	*p* = 1.000	χ^2^ = 3.796	*p* = 0.051	χ^2^ = 403.550	*p* < 0.001
		ICC = 0.853	*p* < 0.001	ICC = 0.730	*p* < 0.001	ICC = 0.562	*p* < 0.001	ICC = 0.206	*p* < 0.001
RE2	1	1162	1157	556	566	709	720	951	1758
	2	2629	2677	3234	3268	3085	3114	2843	2076
		χ^2^ = 0.181	*p* = 0.671	χ^2^ = 0.007	*p* = 0.935	χ^2^ = 0.005	*p* = 0.941	χ^2^ = 358.890	*p* < 0.001
		ICC = 0.863	*p* < 0.001	ICC = 0.724	*p* < 0.001	ICC = 0.591	*p* < 0.001	ICC = 0.289	*p* < 0.001
SF2	1	80	86	38	46	42	66	74	670
	2	309	308	119	134	161	176	231	416
	3	709	720	304	288	402	437	533	575
	4	872	854	558	576	600	621	677	586
	5	1824	1865	2771	2790	2575	2534	2255	1588
		χ^2^ = 0.747	*p* = 0.945	χ^2^ = 2.180	*p* = 0.703	χ^2^ = 7.769	*p* = 0.100	χ^2^ = 653.746	*p* < 0.001
		ICC = 0.880	*p* < 0.001	ICC = 0.771	*p* < 0.001	ICC = 0.661	*p* < 0.001	ICC = 0.183	*p* < 0.001
BP1	1	587	603	1655	1667	1668	1642	1349	1081
	2	528	556	701	744	527	576	524	404
	3	610	602	425	443	427	421	417	416
	4	1353	1349	677	645	784	790	923	1022
	5	610	606	269	263	307	293	445	660
	6	121	118	70	82	92	111	138	251
		χ^2^ = 0.966	*p* = 0.965	χ^2^ = 3.264	*p* = 0.659	χ^2^ = 4.449	*p* = 0.487	χ^2^ = 124.586	*p* < 0.001
		ICC = 0.868	*p* < 0.001	ICC = 0.760	*p* < 0.001	ICC = 0.639	*p* < 0.001	ICC = 0.354	*p* < 0.001
BP2	1	1122	1172	2327	2366	2224	2252	1902	1381
	2	980	955	724	720	701	670	731	614
	3	962	986	444	438	525	543	621	569
	4	559	547	215	210	252	244	382	779
	5	178	174	87	100	98	124	164	491
		χ^2^ = 1.742	*p* = 0.783	χ^2^ = 1.159	*p* = 0.885	χ^2^ = 4.211	*p* = 0.378	χ^2^ = 393.990	*p* < 0.001
		ICC = 0.876	*p* < 0.001	ICC = 0.762	*p* < 0.001	ICC = 0.623	*p* < 0.001	ICC = 0.270	*p* < 0.001
MH1	1	45	48	40	56	39	36	56	325
	2	100	108	78	88	105	126	128	300
	3	272	269	138	150	194	176	236	436
	4	521	529	230	236	281	299	361	381
	5	1140	1118	736	749	770	749	782	786
	6	1739	1762	2599	2555	2429	2449	2251	1606
		χ^2^ = 0.810	*p* = 0.976	χ^2^ = 4.314	*p* = 0.505	χ^2^ = 3.798	*p* = 0.579	χ^2^ = 426.931	*p* < 0.001
		ICC = 0.849	*p* < 0.001	ICC = 0.775	*p* < 0.001	ICC = 0.635	*p* < 0.001	ICC = 0.273	*p* < 0.001
MH4	1	48	48	53	72	56	73	85	330
	2	120	126	104	104	142	174	188	408
	3	268	268	167	181	223	209	281	690
	4	495	487	316	296	400	374	495	438
	5	1306	1326	999	1037	1047	1090	1065	813
	6	1575	1580	2176	2145	1952	1913	1704	1155
		χ^2^ = 0.302	*p* = 0.998	χ^2^ = 4.984	*p* = 0.418	χ^2^ = 8.045	*p* = 0.154	χ^2^ = 540.811	*p* < 0.001
		ICC = 0.851	*p* < 0.001	ICC = 0.780	*p* < 0.001	ICC = 0.673	*p* < 0.001	ICC = 0.277	*p* < 0.001
VT2	1	100	96	604	615	459	422	322	267
	2	417	432	1440	1481	1245	1300	949	602
	3	660	673	792	769	857	867	804	732
	4	1003	1008	458	471	505	487	663	637
	5	1014	992	340	302	472	474	617	780
	6	622	632	189	196	280	284	460	816
		χ^2^ = 0.769	*p* = 0.979	χ^2^ = 3.556	*p* = 0.615	χ^2^ = 3.126	*p* = 0.681	χ^2^ = 204.960	*p* < 0.001
		ICC = 0.884	*p* < 0.001	ICC = 0.781	*p* < 0.001	ICC = 0.671	*p* < 0.001	ICC = 0.435	*p* < 0.001

Frequencies in the cells were estimated by averaging frequencies for each item across the imputations and then rounded to whole numbers. ICC, intraclass correlation coefficient.

**Table 4:** Comparison of actual and imputed SF-6D indices at baseline and follow-ups.

Time Point	Actual		Imputed		MAPE (%)	ICC (95% CI)
	Mean	SE	Mean	SE		
Baseline	0.688	0.0021	0.698	0.0019	4.16	0.814 (0.811, 0.816)
One-year follow-up	0.813	0.0022	0.812	0.0019	6.14	0.682 (0.678, 0.686)
Two-year follow-up	0.796	0.0023	0.797	0.0018	8.15	0.598 (0.592, 0.693)
Five-year follow-up	0.762	0.0025	0.766	0.0018	11.62	0.516 (0.510, 0.522)

SE, standard error; MAPE, mean absolute percentage error; ICC, intraclass correlation coefficient; CI, confidence interval.

## Data Availability

The data that support the study are not publicly available because they contain information that could compromise the privacy and confidentiality of the research participant. The authors will make the data available upon reasonable request and with permission from the Committee of the Scandinavian Obesity Surgery Registry, Sweden.

## Supplementary Materials

The following are available online at https://www.mdpi.com/article/10.3390/ijerph191710827/s1, Table S1: SF-6D domains and the 11 selected SF-36 items that construct the SF-6D domains, Table S2: The short-form-6D (SF-6D), Table S3: Demographic characteristics of the patients in the one-year follow-up, Table S4: Scores for the selected SF-36 items and SF-6D index in the one-year follow-up, Table S5: Demographic characteristics of the patients in the two-year follow-up, Table S6: Scores for the selected SF-36 items and SF-6D index in the two-year follow-up, Table S7: Demographic characteristics of the patients in the five-year follow-up, Table S8: Scores for the selected SF-36 items and SF-6D index in the five-year follow-up, Figure S1: Missingness pattern of the selected 11 SF-36 items in the one-year follow-up for the real-world dataset (red cells indicating missing), Figure S2: Missingness pattern of the selected 11 SF-36 items in the two-year follow-up for the real-world dataset (red cells indicating missing), Figure S3: Missingness pattern of the selected 11 SF-36 items in the five-year follow-up for the real-world dataset (red cells indicating missing), Figure S4: Simulated missingness of the selected 11 SF-36 items in the one-year follow-up for the analytical dataset (red cells indicating missing), Figure S5: Simulated missingness of the selected 11 SF-36 items in the two-year follow-up for the analytical dataset (red cells indicating missing), Figure S6: Simulated missingness of the selected 11 SF-36 items in the five-year follow-up for the analytical dataset (red cells indicating missing).
