# Supplementary material for: Sequential Multiple Imputation for Real-World Health-Related Quality of Life Missing Data after Bariatric Surgery
Source: Int J Environ Res Public Health. 2022 Aug 30;19(17):10827. doi: 10.3390/ijerph191710827 (PMC9518315; doi:10.3390/ijerph191710827)
Supplement: Supplementary file 1 [file ijerph-19-10827-s001.zip › ijerph-1846547-supplementary.pdf]

International Journal of
Environmental Research and Public Health

MDPI

Supplemental materials

Table S1. SF-6D domains and the 11 selected SF-36 items which construct the SF-6D domains

<table>
<tr><th>SF-6D domain</th><th colspan="2">SF-36 item</th></tr>
<tr><td rowspan="3">Physical function</td><td>pf1</td><td>The following items are about activities you might do during a typical day. Does your health now limit you in these activities? If so, how much?
Vigorous activities, such as running, lifting heavy objects, participating in strenuous sports.

1.Yes, limited a lot; 2.Yes, limited a little; 3. No, not limited at all</td></tr>
<tr><td>pf2</td><td>The following items are about activities you might do during a typical day. Does your health now limit you in these activities? If so, how much?
Moderate activities, such as moving a table, pushing a vacuum cleaner, bowling, or playing golf

1.Yes, limited a lot; 2.Yes, limited a little; 3. No, not limited at all</td></tr>
<tr><td>pf10</td><td>The following items are about activities you might do during a typical day. Does your health now limit you in these activities? If so, how much?

Bathing or dressing yourself
1.Yes, limited a lot; 2.Yes, limited a little; 3. No, not limited at all</td></tr>
<tr><td rowspan="2">Role participation</td><td>rp3</td><td>During the past 4 weeks, have you had any of the following problems with your work or other regular daily activities as a result of your physical health?
Were limited in the kind of work or other activities

1.Yes; 2. No</td></tr>
<tr><td>re2</td><td>During the past 4 weeks, have you had any of the following problems with your work or other regular daily activities as a result of any emotional problems (such as feeling depressed or anxious)?
Accomplished less than you would like

1.Yes; 2. No</td></tr>
</table>

**Social function**	**sf2**	During the past 4 weeks, how much of the time has your physical health or emotional problems interfered with your social activities (like visiting with friends, relatives, etc.)<br><br>1. All of the time; 2. Most of the time; 3. Some of the time; 4. A little of the time, 5. None of the time
**Bodily pain**	**bp1**	How much bodily pain have you had during the past 4 weeks?<br><br>1.None; 2. Very mild; 3. Mild; 4. Moderate; 5. Severe; 6. Very severe
**Bodily pain**	**bp2**	During the past 4 weeks, how much did pain interfere with your normal work (including both work outside the home and housework)?<br><br>1.Not at all; 2. A little bit; 3. Moderately; 4. Quite a bit; 5. Extremely
**Mental health**	**mh1**	These questions are about how you feel and how things have been with you during the past 4 weeks. For each question, please give the one answer that comes closest to the way you have been feeling. How much of the time during the past 4 weeks…Have you been a very nervous person?<br><br>1.All of the time; 2. Most of the time; 3. A good bit of the time; 4. Some of the time; 5. A little of the time; 6. None of the time
**Mental health**	**mh4**	These questions are about how you feel and how things have been with you during the past 4 weeks. For each question, please give the one answer that comes closest to the way you have been feeling. How much of the time during the past 4 weeks…Have you felt downhearted and blue?<br><br>1.All of the time; 2. Most of the time; 3. A good bit of the time; 4. Some of the time; 5. A little of the time; 6. None of the time
**Vitality**	**vt2**	These questions are about how you feel and how things have been with you during the past 4 weeks. For each question, please give the one answer that comes closest to the way you have been feeling. How much of the time during the past 4 weeks…Did you have a lot of energy?<br><br>1.All of the time; 2. Most of the time; 3. A good bit of the time; 4. Some of the time; 5. A little of the time; 6. None of the time

Table S2. The short form-6D (SF-6D)[a]

Domain		Severity level
**Physical functioning**		
	1	Your health does not limit you in vigorous activities
	2	Your health limits you a little in vigorous activities
	3	Your health limits you a little in moderate activities
	4	Your health limits you a lot in moderate activities
	5	Your health limits you a little in bathing and dressing
	6	Your health limits you a lot in bathing and dressing
**Role limitations**		
	1	Because of your health, doing your work and other regular activities is not difficult
	2	Because of your health, doing your work and other regular activities is seldom difficult
	3	Because of your health, doing your work and other regular activities is sometimes difficult
	4	Because of your health, doing your work and other regular activities is almost always difficult
	5	Because of your health, doing your work and other regular activities is always difficult
**Social functioning**		
	1	Your health limits your social activities none of the time
	2	Your health limits your social activities a little of the time
	3	Your health limits your social activities some of the time
	4	Your health limits your social activities most of the time
	5	Your health limits your social activities all of the time
**Pain**		
	1	You have no pain
	2	You have pain but it does not interfere with your normal work (both outside the home and housework)
	3	You have pain that interferes with your normal work (both outside the home and housework) a little bit
	4	You have pain that interferes with your normal work (both outside the home and housework) moderately
	5	You have pain that interferes with your normal work (both outside the home and housework) quite a bit
	6	You have pain that interferes with your normal work (both outside the home and housework) extremely
**Mental health**		

	1	You feel tense or downhearted and low none of the time
	2	You feel tense or downhearted and low a little of the time
	3	You feel tense or downhearted and low some of the time
	4	You feel tense or downhearted and low most of the time
	5	You feel tense or downhearted and low all of the time
**Vitality**		
	1	You have a lot of energy all of the time
	2	You have a lot of energy most of the time
	3	You have a lot of energy some of the time
	4	You have a lot of energy a little of the time
	5	You have a lot of energy none of the time

a. The SF-36 items used to construct the SF-6D are as follows: physical function items 1, 2 and 10; role due to physical problems item 3; role limitation due to emotional problems item 2; social function item 2; both bodily pain items; mental health items 1 (alternate version) and 4; and vitality item 2.

Table S3. Demographic characteristics of the patients in year-1 follow-up

Variable		All	Excluded	Analytical dataset	p-value*
n		46753	42796	3957	
BMI (mean (SD))		28.59 (4.57)	28.57 (4.59)	28.70 (4.39)	0.081
Pregnancy (%)	No	9563 (20.5)	8615 (20.1)	948 (24.0)	<0.001
	Yes	382 (0.8)	348 (0.8)	34 (0.9)	
	Missing	36808 (78.7)	33833 (79.1)	2975 (75.2)	
Comorbidity (%)	No	17529 (37.5)	15159 (35.4)	2370 (59.9)	<0.001
	Yes	11636 (24.9)	10069 (23.5)	1567 (39.6)	
	Missing	17588 (37.6)	17568 (41.1)	20 (0.5)	
Sleep apnea (%)	No	28222 (60.4)	24411 (57.0)	3811 (96.3)	<0.001
	Yes	943 (2.0)	817 (1.9)	126 (3.2)	
	Missing	17588 (37.6)	17568 (41.1)	20 (0.5)	
Hypertension (%)	No	24140 (51.6)	21052 (49.2)	3088 (78.0)	<0.001
	Yes	5025 (10.7)	4176 (9.8)	849 (21.5)	
	Missing	17588 (37.6)	17568 (41.1)	20 (0.5)	
Diabetes (%)	No	27812 (59.5)	24096 (56.3)	3716 (93.9)	<0.001
	Yes	1353 (2.9)	1132 (2.6)	221 (5.6)	
	Missing	17588 (37.6)	17568 (41.1)	20 (0.5)	
Dyslipidemia (%)	No	27515 (58.9)	23861 (55.8)	3654 (92.3)	<0.001
	Yes	1650 (3.5)	1367 (3.2)	283 (7.2)	
	Missing	17588 (37.6)	17568 (41.1)	20 (0.5)	
Dyspepsia (%)	No	26985 (57.7)	23309 (54.5)	3676 (92.9)	<0.001
	Yes	2180 (4.7)	1919 (4.5)	261 (6.6)	
	Missing	17588 (37.6)	17568 (41.1)	20 (0.5)	
Diarrhea (%)	No	28790 (61.6)	24903 (58.2)	3887 (98.2)	<0.001
	Yes	375 (0.8)	325 (0.8)	50 (1.3)	
	Missing	17588 (37.6)	17568 (41.1)	20 (0.5)	
Depression (%)	No	25170 (53.8)	21693 (50.7)	3477 (87.9)	<0.001
	Yes	3995 (8.5)	3535 (8.3)	460 (11.6)	
	Missing	17588 (37.6)	17568 (41.1)	20 (0.5)	
Other illness (%)	No	27711 (59.3)	23971 (56.0)	3740 (94.5)	<0.001
	Yes	1480 (3.2)	1283 (3.0)	197 (5.0)	
	Missing	17562 (37.6)	17542 (41.0)	20 (0.5)	
Obesity problem summary score (mean (SD))		18.16 (21.93)	18.45 (22.15)	16.29 (20.39)	<0.001

* Student t-test was used to compare means and chi-squared test was used to compare percentages.

Table S4. Scores for the selected SF-36 items and SF-6D index in year-1 follow-up

SF-6D iterm	Level	All	Excluded	Analytical dataset	p-value*
n		46753	42796	3957	
PF1 (%)	1	3445 (7.4)	2960 (6.9)	485 (12.3)	<0.001
	2	11586 (24.8)	9969 (23.3)	1617 (40.9)	
	3	14114 (30.2)	12284 (28.7)	1830 (46.2)	
	Missing	17608 (37.7)	17583 (41.1)	25 (0.6)	
PF2 (%)	1	1088 (2.3)	968 (2.3)	120 (3.0)	<0.001
	2	3830 (8.2)	3317 (7.8)	513 (13.0)	
	3	24360 (52.1)	21041 (49.2)	3319 (83.9)	
	Missing	17475 (37.4)	17470 (40.8)	5 (0.1)	
PF10 (%)	1	666 (1.4)	606 (1.4)	60 (1.5)	<0.001
	2	1935 (4.1)	1711 (4.0)	224 (5.7)	
	3	26679 (57.1)	23014 (53.8)	3665 (92.6)	
	Missing	17473 (37.4)	17465 (40.8)	8 (0.2)	
RP3 (%)	1	4054 (8.7)	3573 (8.3)	481 (12.2)	<0.001
	2	25035 (53.5)	21589 (50.4)	3446 (87.1)	
	Missing	17664 (37.8)	17634 (41.2)	30 (0.8)	
RE2 (%)	1	5572 (11.9)	4993 (11.7)	579 (14.6)	<0.001
	2	23415 (50.1)	20082 (46.9)	3333 (84.2)	
	Missing	17766 (38.0)	17721 (41.4)	45 (1.1)	
SF2 (%)	1	395 (0.8)	352 (0.8)	43 (1.1)	<0.001
	2	1253 (2.7)	1128 (2.6)	125 (3.2)	
	3	2899 (6.2)	2581 (6.0)	318 (8.0)	
	4	4522 (9.7)	3946 (9.2)	576 (14.6)	
	5	19873 (42.5)	17028 (39.8)	2845 (71.9)	
	Missing	17811 (38.1)	17761 (41.5)	50 (1.3)	
BP1 (%)	1	12830 (27.4)	11128 (26.0)	1702 (43.0)	<0.001
	2	4981 (10.7)	4258 (9.9)	723 (18.3)	
	3	3349 (7.2)	2908 (6.8)	441 (11.1)	
	4	5000 (10.7)	4301 (10.1)	699 (17.7)	
	5	2274 (4.9)	1992 (4.7)	282 (7.1)	
	6	685 (1.5)	612 (1.4)	73 (1.8)	
	Missing	17634 (37.7)	17597 (41.1)	37 (0.9)	
BP2 (%)	1	17574 (37.6)	15176 (35.5)	2398 (60.6)	<0.001
	2	5566 (11.9)	4816 (11.3)	750 (19.0)	
	3	3390 (7.3)	2936 (6.9)	454 (11.5)	
	4	1785 (3.8)	1562 (3.6)	223 (5.6)	
	5	798 (1.7)	704 (1.6)	94 (2.4)	
	Missing	17640 (37.7)	17602 (41.1)	38 (1.0)	
MH1 (%)	1	382 (0.8)	338 (0.8)	44 (1.1)	<0.001

	2	772 (1.7)	689 (1.6)	83 (2.1)	
	3	1468 (3.1)	1321 (3.1)	147 (3.7)	
	4	2357 (5.0)	2121 (5.0)	236 (6.0)	
	5	6274 (13.4)	5514 (12.9)	760 (19.2)	
	6	17982 (38.5)	15309 (35.8)	2673 (67.6)	
	Missing	17518 (37.5)	17504 (40.9)	14 (0.4)	
MH4 (%)	1	421 (0.9)	366 (0.9)	55 (1.4)	<0.001
	2	1026 (2.2)	914 (2.1)	112 (2.8)	
	3	1694 (3.6)	1520 (3.6)	174 (4.4)	
	4	2843 (6.1)	2522 (5.9)	321 (8.1)	
	5	8108 (17.3)	7073 (16.5)	1035 (26.2)	
	6	15099 (32.3)	12859 (30.0)	2240 (56.6)	
	Missing	17562 (37.6)	17542 (41.0)	20 (0.5)	
VT2 (%)	1	4049 (8.7)	3420 (8.0)	629 (15.9)	<0.001
	2	10128 (21.7)	8651 (20.2)	1477 (37.3)	
	3	6227 (13.3)	5412 (12.6)	815 (20.6)	
	4	3996 (8.5)	3524 (8.2)	472 (11.9)	
	5	3057 (6.5)	2708 (6.3)	349 (8.8)	
	6	1756 (3.8)	1555 (3.6)	201 (5.1)	
	Missing	17540 (37.5)	17526 (41.0)	14 (0.4)	
Index (mean (SD))		0.80 (0.14)	0.80 (0.14)	0.81 (0.13)	<0.001

[*] Student t-test was used to compare means and chi-squared test was used to compare percentages.

Table S5. Demographic characteristics of the patients in year-2 follow-up

Variable		All	Excluded	Analytical dataset	p-value[*]
n		46753	42796	3957	
BMI (mean (SD))		28.45 (4.68)	28.48 (4.74)	28.35 (4.45)	0.122
Pregnancy (%)	No	7276 (15.6)	5860 (13.7)	1416 (35.8)	<0.001
	Yes	279 (0.6)	221 (0.5)	58 (1.5)	
	Missing	39198 (83.8)	36715 (85.8)	2483 (62.7)	
Comorbidity (%)	No	10590 (22.7)	8282 (19.4)	2308 (58.3)	<0.001
	Yes	7689 (16.4)	6087 (14.2)	1602 (40.5)	
	Missing	28474 (60.9)	28427 (66.4)	47 (1.2)	
Sleep apnea (%)	No	17741 (37.9)	13929 (32.5)	3812 (96.3)	<0.001
	Yes	538 (1.2)	440 (1.0)	98 (2.5)	
	Missing	28474 (60.9)	28427 (66.4)	47 (1.2)	
Hypertension (%)	No	14889 (31.8)	11800 (27.6)	3089 (78.1)	<0.001
	Yes	3390 (7.3)	2569 (6.0)	821 (20.7)	
	Missing	28474 (60.9)	28427 (66.4)	47 (1.2)	
Diabetes (%)	No	17303 (37.0)	13616 (31.8)	3687 (93.2)	<0.001
	Yes	976 (2.1)	753 (1.8)	223 (5.6)	
	Missing	28474 (60.9)	28427 (66.4)	47 (1.2)	
Dyslipidemia (%)	No	17153 (36.7)	13507 (31.6)	3646 (92.1)	<0.001
	Yes	1126 (2.4)	862 (2.0)	264 (6.7)	
	Missing	28474 (60.9)	28427 (66.4)	47 (1.2)	
Dyspepsia (%)	No	16710 (35.7)	13077 (30.6)	3633 (91.8)	<0.001
	Yes	1569 (3.4)	1292 (3.0)	277 (7.0)	
	Missing	28474 (60.9)	28427 (66.4)	47 (1.2)	
Diarrhea (%)	No	17959 (38.4)	14126 (33.0)	3833 (96.9)	<0.001
	Yes	320 (0.7)	243 (0.6)	77 (1.9)	
	Missing	28474 (60.9)	28427 (66.4)	47 (1.2)	
Depression (%)	No	15542 (33.2)	12139 (28.4)	3403 (86.0)	<0.001
	Yes	2737 (5.9)	2230 (5.2)	507 (12.8)	
	Missing	28474 (60.9)	28427 (66.4)	47 (1.2)	
Other illness (%)	No	17563 (37.6)	13848 (32.4)	3715 (93.9)	<0.001
	Yes	769 (1.6)	572 (1.3)	197 (5.0)	
	Missing	28421 (60.8)	28376 (66.3)	45 (1.1)	
Obesity problem summary score (mean (SD))		20.53 (24.41)	21.04 (24.78)	18.63 (22.90)	<0.001

[*] Student t-test was used to compare means and chi-squared test was used to compare percentages.

Table S6. Scores for the selected SF-36 items and SF-6D index in year-2 follow-up

SF-6D iterm	Level	All	Excluded	Analytical dataset	p-value*
n		46753	42796	3957	
PF1 (%)	1	2593 (5.5)	2064 (4.8)	529 (13.4)	<0.001
	2	6808 (14.6)	5354 (12.5)	1454 (36.7)	
	3	9093 (19.4)	7149 (16.7)	1944 (49.1)	
	Missing	28259 (60.4)	28229 (66.0)	30 (0.8)	
PF2 (%)	1	727 (1.6)	607 (1.4)	120 (3.0)	<0.001
	2	2766 (5.9)	2207 (5.2)	559 (14.1)	
	3	15070 (32.2)	11810 (27.6)	3260 (82.4)	
	Missing	28190 (60.3)	28172 (65.8)	18 (0.5)	
PF10 (%)	1	497 (1.1)	414 (1.0)	83 (2.1)	<0.001
	2	1495 (3.2)	1188 (2.8)	307 (7.8)	
	3	16577 (35.5)	13018 (30.4)	3559 (89.9)	
	Missing	28184 (60.3)	28176 (65.8)	8 (0.2)	
RP3 (%)	1	3077 (6.6)	2509 (5.9)	568 (14.4)	<0.001
	2	15371 (32.9)	12018 (28.1)	3353 (84.7)	
	Missing	28305 (60.5)	28269 (66.1)	36 (0.9)	
RE2 (%)	1	4103 (8.8)	3368 (7.9)	735 (18.6)	<0.001
	2	14286 (30.6)	11109 (26.0)	3177 (80.3)	
	Missing	28364 (60.7)	28319 (66.2)	45 (1.1)	
SF2 (%)	1	321 (0.7)	276 (0.6)	45 (1.1)	<0.001
	2	1016 (2.2)	845 (2.0)	171 (4.3)	
	3	2310 (4.9)	1889 (4.4)	421 (10.6)	
	4	2991 (6.4)	2375 (5.5)	616 (15.6)	
	5	11720 (25.1)	9072 (21.2)	2648 (66.9)	
	Missing	28395 (60.7)	28339 (66.2)	56 (1.4)	
BP1 (%)	1	7828 (16.7)	6119 (14.3)	1709 (43.2)	<0.001
	2	2702 (5.8)	2149 (5.0)	553 (14.0)	
	3	2109 (4.5)	1670 (3.9)	439 (11.1)	
	4	3616 (7.7)	2814 (6.6)	802 (20.3)	
	5	1644 (3.5)	1320 (3.1)	324 (8.2)	
	6	550 (1.2)	453 (1.1)	97 (2.5)	
	Missing	28304 (60.5)	28271 (66.1)	33 (0.8)	
BP2 (%)	1	10551 (22.6)	8257 (19.3)	2294 (58.0)	<0.001
	2	3411 (7.3)	2693 (6.3)	718 (18.1)	
	3	2552 (5.5)	2013 (4.7)	539 (13.6)	
	4	1313 (2.8)	1050 (2.5)	263 (6.6)	
	5	651 (1.4)	545 (1.3)	106 (2.7)	
	Missing	28275 (60.5)	28238 (66.0)	37 (0.9)	
MH1 (%)	1	286 (0.6)	244 (0.6)	42 (1.1)	<0.001

	2	549 (1.2)	440 (1.0)	109 (2.8)	
	3	1138 (2.4)	935 (2.2)	203 (5.1)	
	4	1654 (3.5)	1364 (3.2)	290 (7.3)	
	5	3900 (8.3)	3097 (7.2)	803 (20.3)	
	6	11020 (23.6)	8527 (19.9)	2493 (63.0)	
	Missing	28206 (60.3)	28189 (65.9)	17 (0.4)	
MH4 (%)	1	347 (0.7)	286 (0.7)	61 (1.5)	<0.001
	2	887 (1.9)	736 (1.7)	151 (3.8)	
	3	1320 (2.8)	1091 (2.5)	229 (5.8)	
	4	2060 (4.4)	1649 (3.9)	411 (10.4)	
	5	5130 (11.0)	4049 (9.5)	1081 (27.3)	
	6	8791 (18.8)	6782 (15.8)	2009 (50.8)	
	Missing	28218 (60.4)	28203 (65.9)	15 (0.4)	
VT2 (%)	1	2065 (4.4)	1582 (3.7)	483 (12.2)	<0.001
	2	5635 (12.1)	4353 (10.2)	1282 (32.4)	
	3	3882 (8.3)	3003 (7.0)	879 (22.2)	
	4	2763 (5.9)	2248 (5.3)	515 (13.0)	
	5	2604 (5.6)	2117 (4.9)	487 (12.3)	
	6	1588 (3.4)	1294 (3.0)	294 (7.4)	
	Missing	28216 (60.4)	28199 (65.9)	17 (0.4)	
Index (mean (SD))		0.78 (0.15)	0.78 (0.15)	0.80 (0.14)	<0.001

* Student t-test was used to compare means and chi-squared test was used to compare percentages.

Table S7. Demographic characteristics of the patients in year-5 follow-up

Variable		All	Excluded	Analytical dataset	p-value*
n		46753	42796	3957	
BMI (mean (SD))		29.91 (4.97)	29.94 (4.96)	29.89 (4.98)	0.655
Pregnancy (%)	No	3214 (6.9)	1673 (3.9)	1541 (38.9)	<0.001
	Yes	83 (0.2)	39 (0.1)	44 (1.1)	
	Missing	43456 (92.9)	41084 (96.0)	2372 (59.9)	
Comorbidity (%)	No	4014 (8.6)	2018 (4.7)	1996 (50.4)	<0.001
	Yes	3749 (8.0)	1878 (4.4)	1871 (47.3)	
	Missing	38990 (83.4)	38900 (90.9)	90 (2.3)	
Sleep apnea (%)	No	7567 (16.2)	3801 (8.9)	3766 (95.2)	<0.001
	Yes	196 (0.4)	95 (0.2)	101 (2.6)	
	Missing	38990 (83.4)	38900 (90.9)	90 (2.3)	
Hypertension (%)	No	6059 (13.0)	3080 (7.2)	2979 (75.3)	<0.001
	Yes	1704 (3.6)	816 (1.9)	888 (22.4)	
	Missing	38990 (83.4)	38900 (90.9)	90 (2.3)	
Diabetes (%)	No	7246 (15.5)	3628 (8.5)	3618 (91.4)	<0.001
	Yes	517 (1.1)	268 (0.6)	249 (6.3)	
	Missing	38990 (83.4)	38900 (90.9)	90 (2.3)	
Dyslipidemia (%)	No	7224 (15.5)	3663 (8.6)	3561 (90.0)	<0.001
	Yes	539 (1.2)	233 (0.5)	306 (7.7)	
	Missing	38990 (83.4)	38900 (90.9)	90 (2.3)	
Dyspepsia (%)	No	7036 (15.0)	3551 (8.3)	3485 (88.1)	<0.001
	Yes	727 (1.6)	345 (0.8)	382 (9.7)	
	Missing	38990 (83.4)	38900 (90.9)	90 (2.3)	
Diarrhea (%)	No	7532 (16.1)	3766 (8.8)	3766 (95.2)	<0.001
	Yes	231 (0.5)	130 (0.3)	101 (2.6)	
	Missing	38990 (83.4)	38900 (90.9)	90 (2.3)	
Depression (%)	No	6388 (13.7)	3192 (7.5)	3196 (80.8)	<0.001
	Yes	1375 (2.9)	704 (1.6)	671 (17.0)	
	Missing	38990 (83.4)	38900 (90.9)	90 (2.3)	
Other illness (%)	No	7483 (16.0)	3767 (8.8)	3716 (93.9)	<0.001
	Yes	329 (0.7)	160 (0.4)	169 (4.3)	
	Missing	38941 (83.3)	38869 (90.8)	72 (1.8)	
Obesity problem summary score (mean (SD))		23.89 (26.96)	25.60 (27.73)	22.16 (26.05)	<0.001

* Student t-test was used to compare means and chi-squared test was used to compare percentages.

Table S8. Scores for the selected SF-36 items and SF-6D index in year-5 follow-up

SF-6D iterm	Level	All	Excluded	Analytical dataset	p-value*
n		46753	42796	3957	
PF1 (%)	1	1692 (3.6)	908 (2.1)	784 (19.8)	<0.001
	2	3098 (6.6)	1543 (3.6)	1555 (39.3)	
	3	3163 (6.8)	1561 (3.6)	1602 (40.5)	
	Missing	38800 (83.0)	38784 (90.6)	16 (0.4)	
PF2 (%)	1	432 (0.9)	267 (0.6)	165 (4.2)	<0.001
	2	1598 (3.4)	854 (2.0)	744 (18.8)	
	3	5935 (12.7)	2904 (6.8)	3031 (76.6)	
	Missing	38788 (83.0)	38771 (90.6)	17 (0.4)	
PF10 (%)	1	201 (0.4)	112 (0.3)	89 (2.2)	<0.001
	2	816 (1.7)	429 (1.0)	387 (9.8)	
	3	6951 (14.9)	3482 (8.1)	3469 (87.7)	
	Missing	38785 (83.0)	38773 (90.6)	12 (0.3)	
RP3 (%)	1	1784 (3.8)	972 (2.3)	812 (20.5)	<0.001
	2	6110 (13.1)	3005 (7.0)	3105 (78.5)	
	Missing	38859 (83.1)	38819 (90.7)	40 (1.0)	
RE2 (%)	1	2146 (4.6)	1157 (2.7)	989 (25.0)	<0.001
	2	5746 (12.3)	2822 (6.6)	2924 (73.9)	
	Missing	38861 (83.1)	38817 (90.7)	44 (1.1)	
SF2 (%)	1	196 (0.4)	120 (0.3)	76 (1.9)	<0.001
	2	577 (1.2)	337 (0.8)	240 (6.1)	
	3	1230 (2.6)	671 (1.6)	559 (14.1)	
	4	1406 (3.0)	708 (1.7)	698 (17.6)	
	5	4457 (9.5)	2145 (5.0)	2312 (58.4)	
	Missing	38887 (83.2)	38815 (90.7)	72 (1.8)	
BP1 (%)	1	2737 (5.9)	1355 (3.2)	1382 (34.9)	<0.001
	2	1043 (2.2)	504 (1.2)	539 (13.6)	
	3	862 (1.8)	428 (1.0)	434 (11.0)	
	4	1933 (4.1)	976 (2.3)	957 (24.2)	
	5	1028 (2.2)	567 (1.3)	461 (11.7)	
	6	313 (0.7)	170 (0.4)	143 (3.6)	
	Missing	38837 (83.1)	38796 (90.7)	41 (1.0)	
BP2 (%)	1	3809 (8.1)	1862 (4.4)	1947 (49.2)	<0.001
	2	1525 (3.3)	764 (1.8)	761 (19.2)	
	3	1347 (2.9)	698 (1.6)	649 (16.4)	
	4	862 (1.8)	472 (1.1)	390 (9.9)	
	5	376 (0.8)	203 (0.5)	173 (4.4)	
	Missing	38834 (83.1)	38797 (90.7)	37 (0.9)	
MH1 (%)	1	137 (0.3)	75 (0.2)	62 (1.6)	<0.001

	2	308 (0.7)	170 (0.4)	138 (3.5)	
	3	550 (1.2)	306 (0.7)	244 (6.2)	
	4	759 (1.6)	388 (0.9)	371 (9.4)	
	5	1652 (3.5)	845 (2.0)	807 (20.4)	
	6	4547 (9.7)	2234 (5.2)	2313 (58.5)	
	Missing	38800 (83.0)	38778 (90.6)	22 (0.6)	
MH4 (%)	1	199 (0.4)	110 (0.3)	89 (2.2)	<0.001
	2	456 (1.0)	260 (0.6)	196 (5.0)	
	3	649 (1.4)	358 (0.8)	291 (7.4)	
	4	1052 (2.3)	546 (1.3)	506 (12.8)	
	5	2225 (4.8)	1119 (2.6)	1106 (28.0)	
	6	3367 (7.2)	1618 (3.8)	1749 (44.2)	
	Missing	38805 (83.0)	38785 (90.6)	20 (0.5)	
VT2 (%)	1	637 (1.4)	303 (0.7)	334 (8.4)	<0.001
	2	1864 (4.0)	887 (2.1)	977 (24.7)	
	3	1594 (3.4)	770 (1.8)	824 (20.8)	
	4	1392 (3.0)	709 (1.7)	683 (17.3)	
	5	1418 (3.0)	778 (1.8)	640 (16.2)	
	6	1040 (2.2)	562 (1.3)	478 (12.1)	
	Missing	38808 (83.0)	38787 (90.6)	21 (0.5)	
Index (mean (SD))		0.75 (0.15)	0.74 (0.15)	0.76 (0.15)	<0.001

* Student t-test was used to compare means and chi-squared test was used to compare percentages.

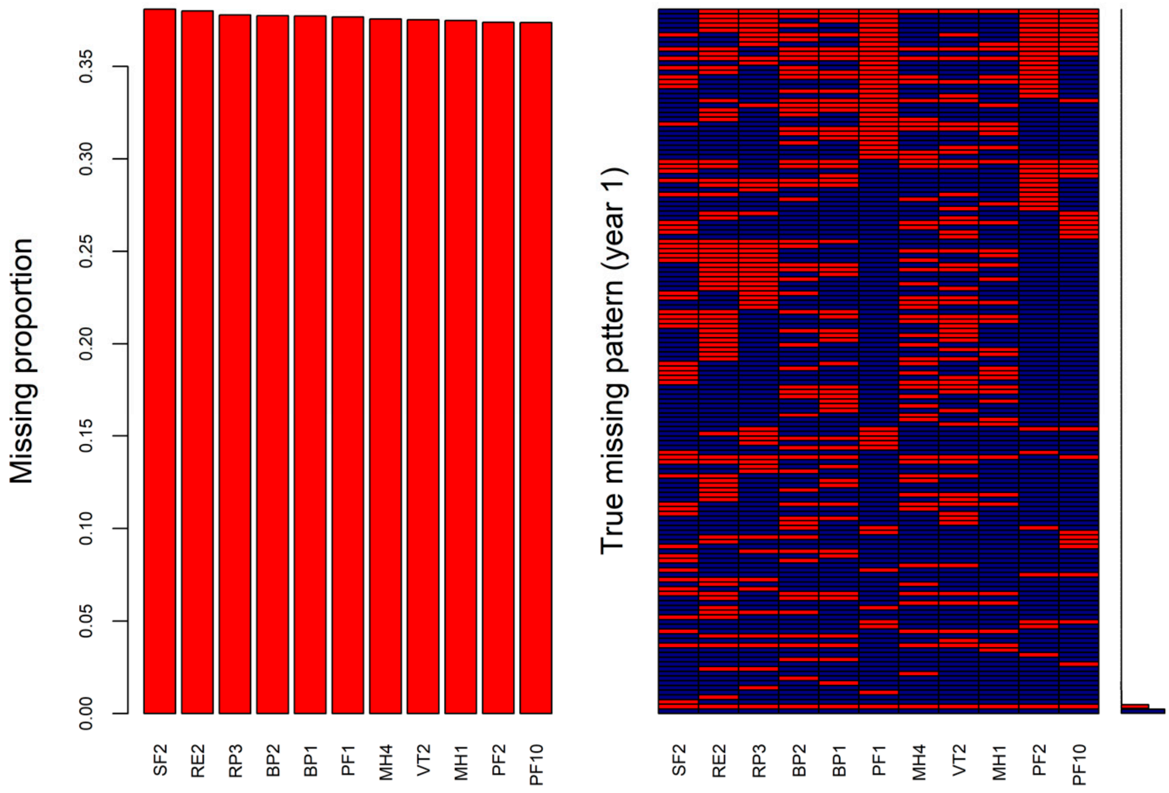

Figure S1. Missingness pattern of the selected 11 SF-36 items in year-1 follow-up for the real-world dataset (red cells indicating missing)

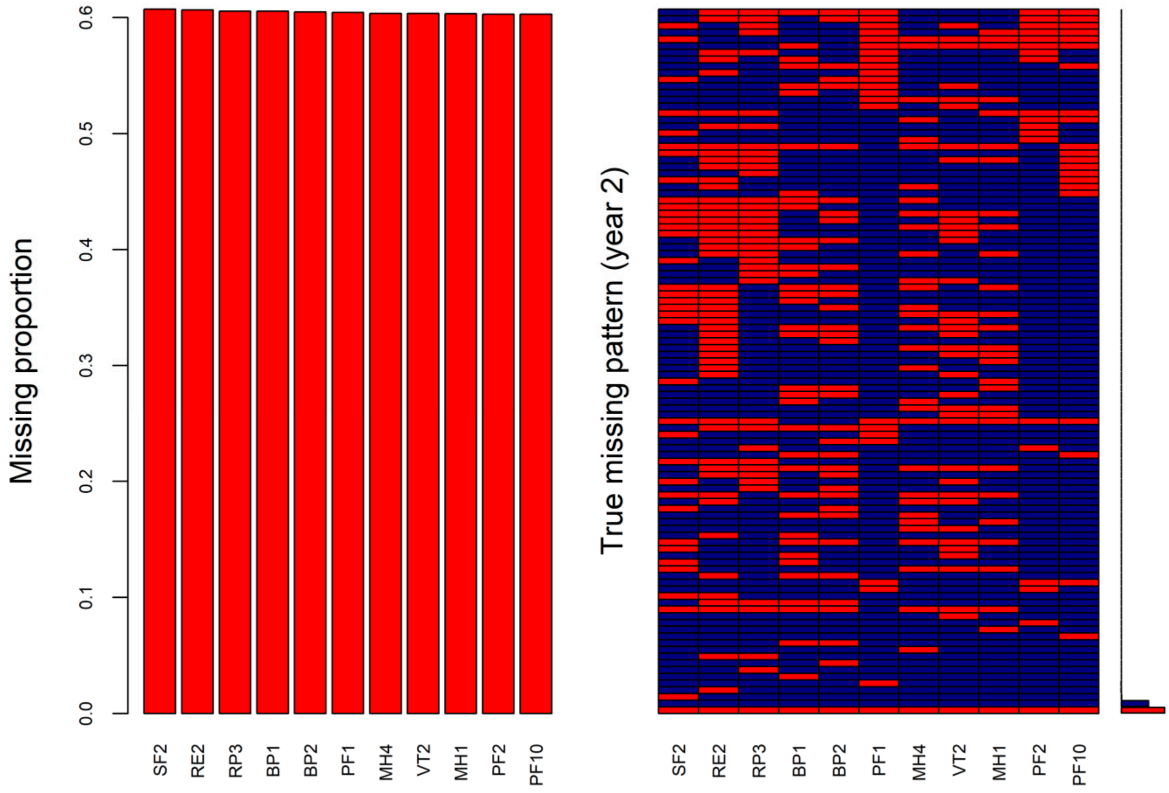

Figure S2. Missingness pattern of the selected 11 SF-36 items in year-2 follow-up for the real-world dataset (red cells indicating missing)

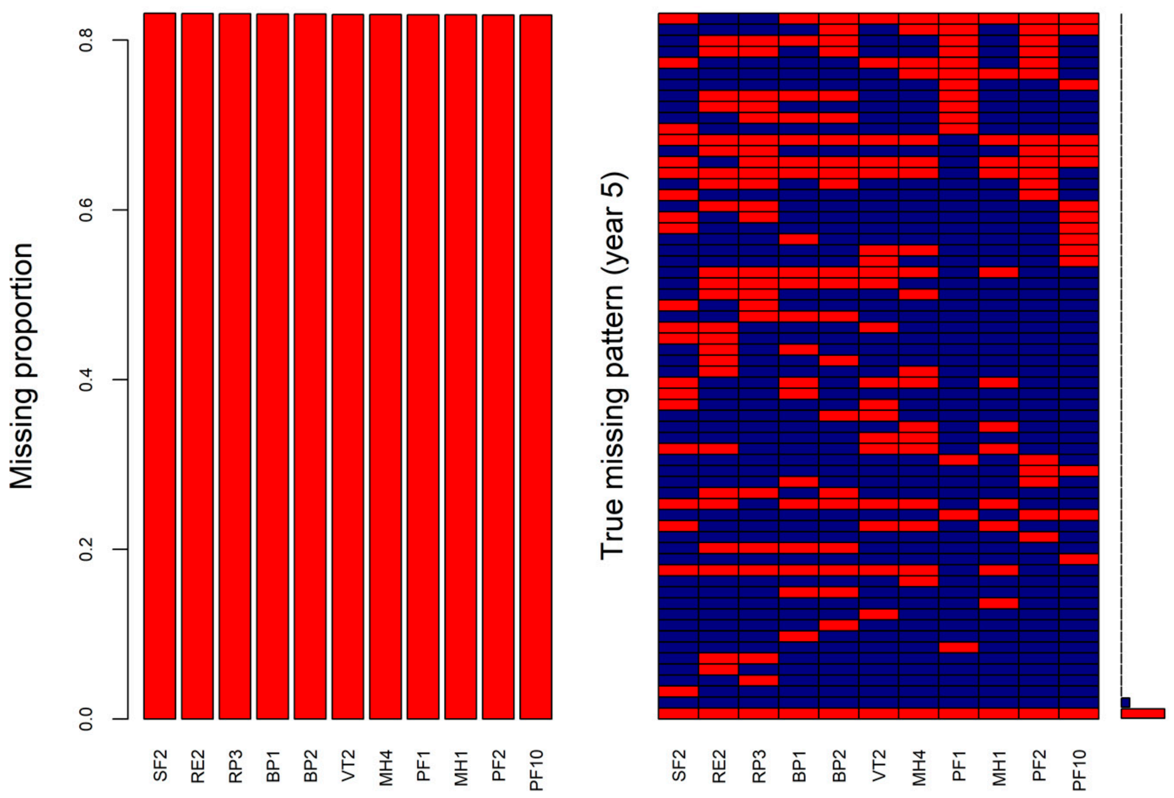

Figure S3. Missingness pattern of the selected 11 SF-36 items in year-5 follow-up for the real-world dataset (red cells indicating missing)

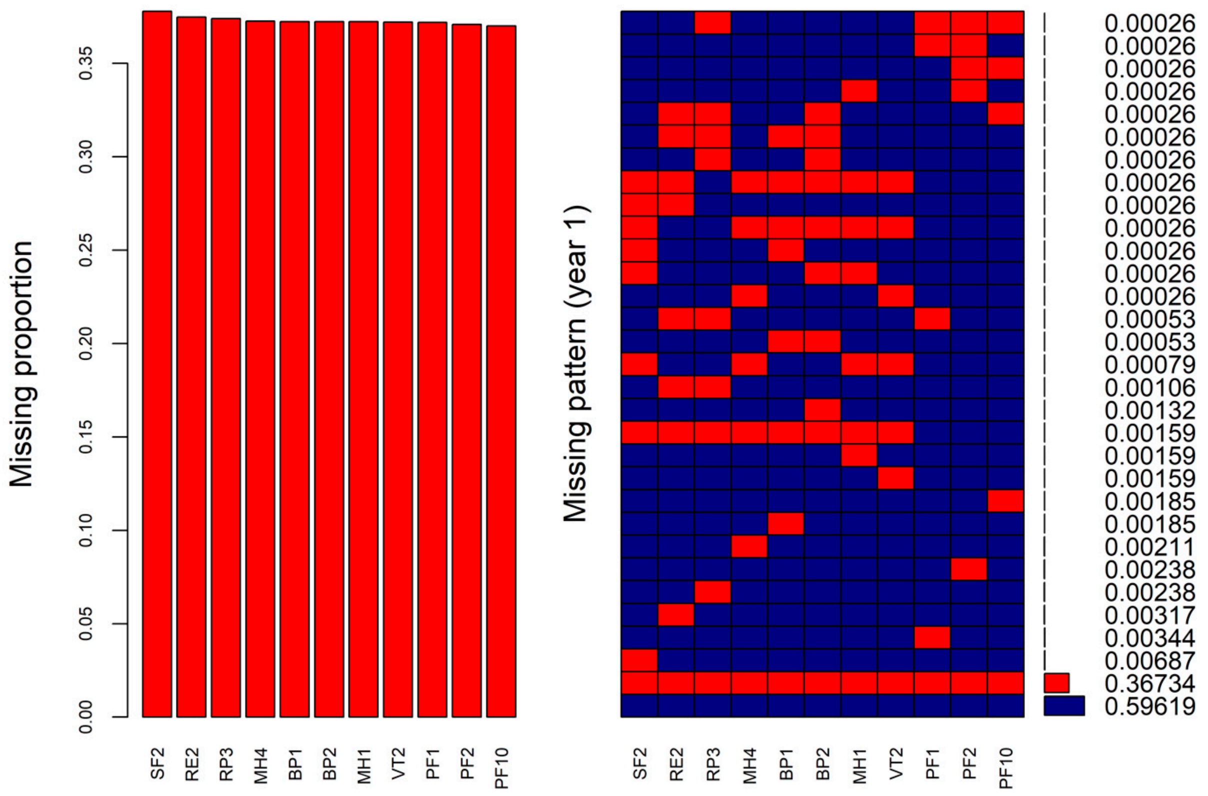

Figure S4. Simulated missingness of the selected 11 SF-36 items in year-1 follow-up for the analytical dataset (red cells indicating missing)

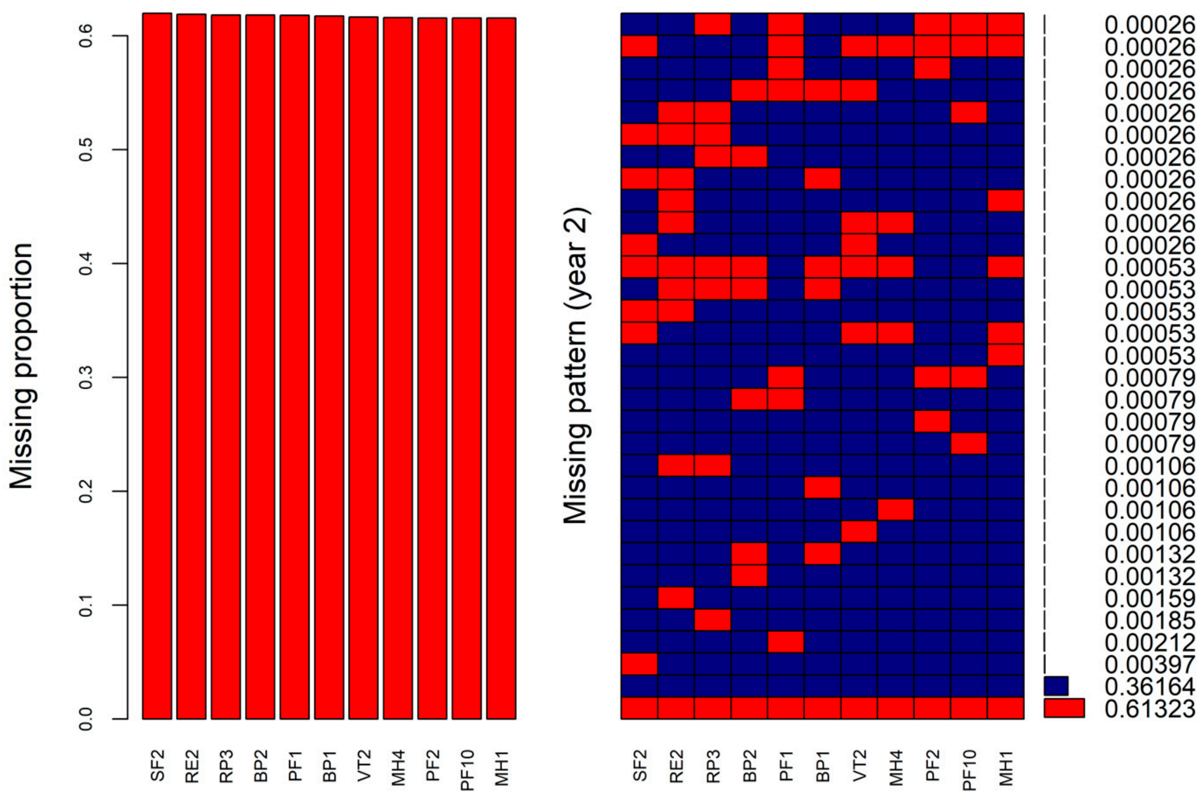

Figure S5. Simulated missingness of the selected 11 SF-36 items in year-2 follow-up for the analytical dataset (red cells indicating missing)

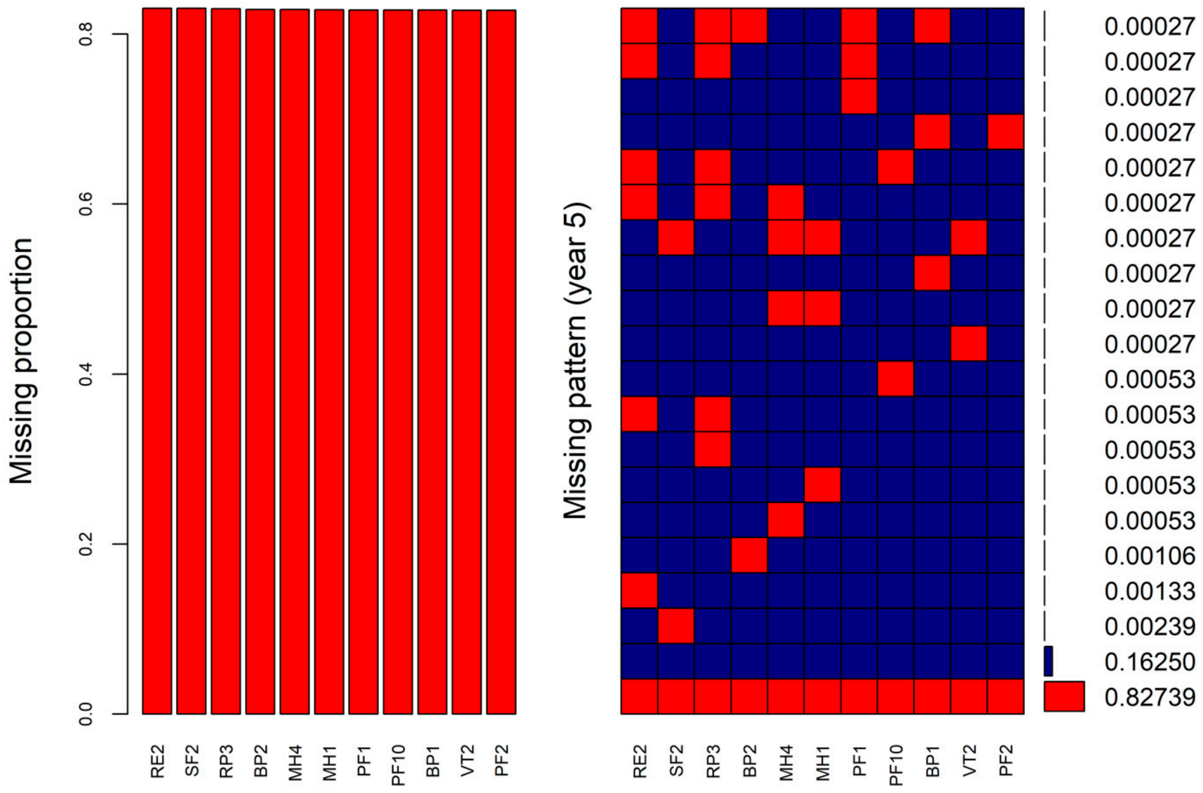

Figure S6. Simulated missingness of the selected 11 SF-36 items in year-5 follow-up for the analytical dataset (red cells indicating missing).
